# 

**Published:** 1868-05-01

**Authors:** 


### CHICAGO MEDICAL JOURNAL.

**Edited by J. ADAMS ALLEN, M.D., LL.D.,**

Professor of Principles and Practice of Medicine and Clinical Medicine in Rush Medical College.

All letters for the JOURNAL should be addressed to Dr. J. ADAMS ALLEN P. O. Box 1948, Chicago, Ill.

TERMS — $3 Per Annum, in Advance;

$4 it not paid before 1st of July, in each year. Single copies, 25 cents.

### *Illinois State Medical Society.*

The annual session of the society occurs, the present year, the *third Tuesday in May*. The place of meeting has been fixed at Quincy, in Adams county. As this point is easily accessible by railroad from all parts of the State, is a beautiful city, its citizens hospitable, and its medical corps enthusiastic and of a high order of talent, a more desirable locality could scarcely have been selected. A very agreeable time may confidently be anticipated. Many subjects of great importance were laid over last year for consideration at this meeting, and it is hoped there will be an unusually large attendance. The fruit and vegetable questions having been exhausted by the elaborate and frequent speeches of our friend J. last year—the sphygmograph having proved that three fingers of Bourbon produce the same effect as three weeks of typhoid fever—the Chicago Sanitary Board having cleaned the city of chances of cholera, there will remain every opportunity to "elevate the profession by appeals for legislative help therefor," or by resolving that the number of graduated practitioners shall be restricted by Utopian regulations—or, which perhaps would be a more feasible, common sense and compendious way, by discussion of strictly scientific medical subjects, of which there are an abundance in waiting.

Let the city of Quincy this year, at all events, be a professional Mecca; or a Jerusalem, to which the tribes shall go up, each man to be taxed according to his mental ability.

